# Hall Amplifier Nanoscale Device (HAND): Modeling, Simulations and Feasibility Analysis for THz Sensor

**DOI:** 10.3390/nano9111618

**Published:** 2019-11-14

**Authors:** Avi Karsenty, Raz Mottes

**Affiliations:** 1 Advanced Laboratory of Electro-Optics (ALEO), Department of Applied Physics/Electro-Optics Engineering, Lev Academic Center, Jerusalem 9116001, Israel; rm1995ex@gmail.com; 2 Nanotechnology Center for Education and Research, Lev Academic Center, Jerusalem 9116001, Israel

**Keywords:** Hall Effect, Hall Amplifier, nanoscale sensor, modeling, simulations, heat effects

## Abstract

HAND (Hall Amplifier Nanoscale Device), a new nano-metric device, was designed, simulated, and modeled for feasibility analysis, with the challenge of combining a well-known macro effect into the nanoscale world. HAND is based on the well-known Hall Effect, and it may enable circuitry working at very high frequencies (tens of Tera-Hertz). The architecture, design, and simulations were performed while using Comsol Multi-Physics Package Software. Complementary accurate analytical models were developed to support the understanding of the device functionality, including treatment of specific phenomena, such as heat transfer, and mega-magnet feasibility inside integrated circuits. This new device, combining both the Hall Effect and nanoscale dimensions, holds the potential to change the computing rates in the microelectronics circuitry world, and can serve as a game changer.

## 1. Introduction

The Hall Amplifier is a well-known and established phenomenon since it was discovered by Edwin Hall in 1879 [1]. The Hall Effect is the production of a voltage difference (the Hall Voltage) across an electrical conductor, transverse to an electric current in the conductor, and to an applied magnetic field perpendicular to the current. As electrons travel through the conductor that lies in a magnetic Field $\vec{B}$, the electrons will experience a magnetic force. This magnetic force will cause the electrons to travel closer to one side than the other, creating a negative charge on one side and a positive on the other. This separation of charge creates a voltage difference that is known as the Hall Voltage. Electrons move in the Y direction and an electric field component appears in the Y direction E_Y_. This will continue until the Lorentz force is equal and opposite to the electric force due to the buildup of electrons—that is, a steady condition arises. The voltage builds up until the electric field produces an electric force on the charges that is equal and opposite of the magnetic force. This effect is known as the Hall Effect, which can be used to distinguish the currents that are composed of positively charged particles from those that are composed of negatively charged particles, as emphasized by Lorentz [2]. With time, the idea of using and moving the Hall Effect from the macro to micro and nano scales caught the attention of various research initiatives. Several types of devices sharing Hall Effect were studied thoroughly in the past decade, such as MoS_2_ transistors [3] and Hall Effect sensors [4]. Moving forward with electromagnetic fields in circuitry, Ballistic deflection transistors were also studied [5]. In those cases, the ballistic effects in transistors were also examined [6,7,8].

As mentioned above, the notion of designing a Hall Amplifier is not new and it has already existed for six decades [9,10,11]. In fact, a double challenge exists: Time review and Technology capability. From the time point of view, the idea of Hall Effect amplifiers seems to be hidden in very old and hardly accessible papers, as cited above. From the technology point of view, the implementation of these early ideas has been difficult or even impossible due to the lack of suitable materials and technologies. Such materials and technologies are currently emerging and, therefore, a re-examination of the entire concept appears mandatory.

Our innovation is to try to design a nanoscale device that can be integrated in circuits. The goal is to find out whether the existing nanotechnology and contemporary capability to make extremely small and complex devices can be used to advance the state of art in high frequency electronics. In particular, our goal is to obtain amplifiers and sources in THz frequencies. Moreover, and in contrast to regular transistors that work with voltage as input and with current as output, the Hall Amplifier works differently—i.e., with current as an input and voltage as output. This characteristic could lead to different applications, and if such a device is achieved it would indeed be revolutionary. We explore an alternative with magnetic fields contrary to past research of ballistic deflation devices in which the deflection was conducted with an electrical field. This article presents the architecture, design, modeling, and simulations of a Hall Amplifier Nanoscale Device, the HAND.

## 2. Device Structure

### 2.1. Nanoscale Device Concept and Advantages

**Figure 1:**
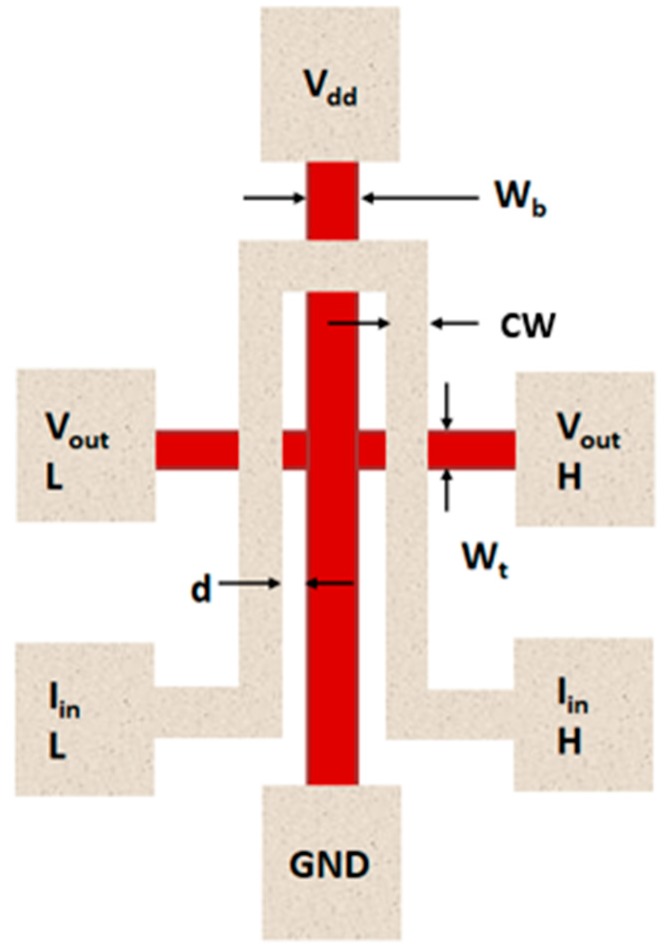
Proposed structure and parameter names of the Hall Amplifier.

Starting from the schematic design of the Hall Amplifier device, as presented in Figure 1, we progressed with a translation of the concept into a well-defined structure in numerical application. The structure of the device is made of a Hall Bar and of an electro magnet close to it. By parameterizing the simulation dimensions, the bar voltage and current, it would be easier to study different parameters and their physical effect on the Hall Bar as a trans-impedance amplifier and its frequency response. The expected outcome of such a study is to analyze the materials and dimensions that should be used to make this device operational at THz frequencies. Undoubtedly, a successful accomplishment of this challenge can enable clear nano-technological advantages. Operating the Hall Bar at AC conditions and measuring output power was done before, but the real question is whether nanotechnology can be used to actually produce a device from this concept. It is not a simple question to answer, however using a simulation environment seems to be very helpful.

### 2.2. Device Architecture and Design

**Figure 2:**
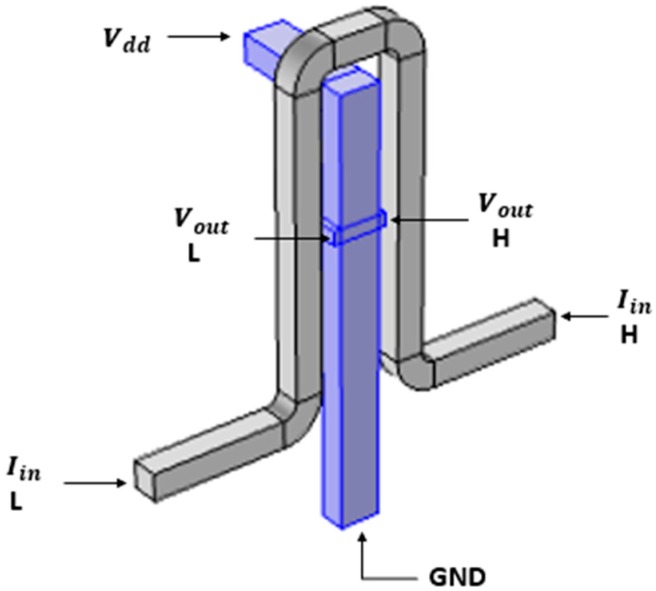
Materials composing the design.

The design of the HAND consists of copper coil surrounding the Hall Bar, which is made of doped GaAs, as shown in Figure 2. In more advanced simulations, and after other materials like silicon were tested, the Hall Bar was chosen to be made of doped GaAs because it has high mobility. Comsol Multi-Physics uses the Finite Element method [12,13], which is a numerical method for solving partial differential equations by domain discretization.

**Figure 3:**
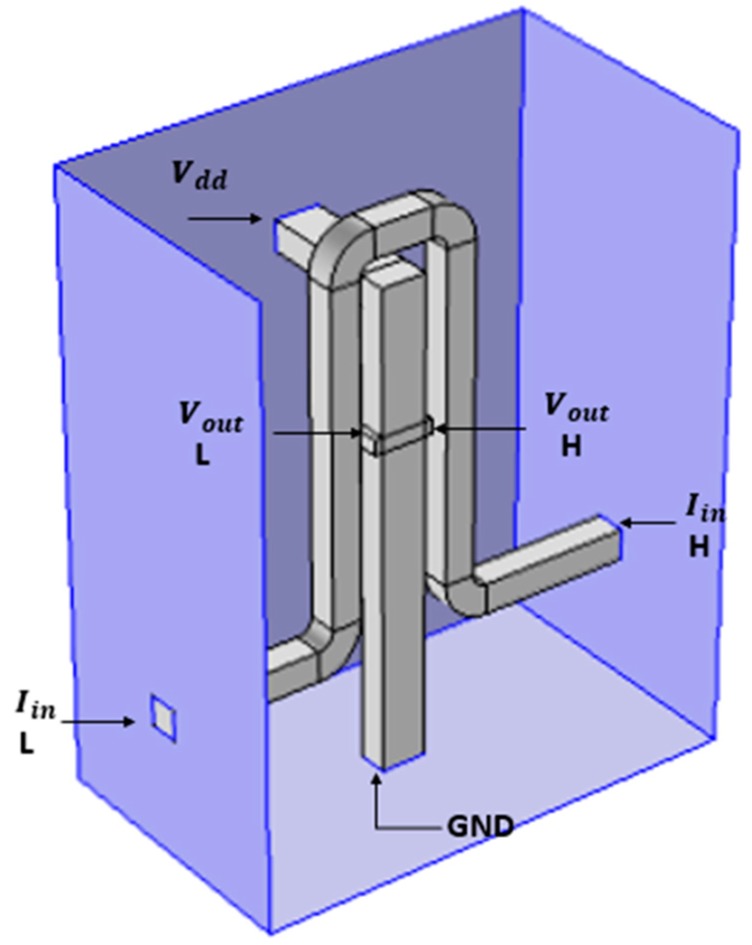
Hall Bar geometry for the simulations.

As part of HAND’s preliminary design, a bent rear bar shape was chosen for boundary conditions and smooth and convenient usage, when simulating with the Comsol software package. HAND’s final shape towards its fabrication will be optimized according to process guidelines and limitations. Figure 3 presents the design of the device for simulation purpose. The main concept is to run electric current through the coil which surrounds the Hall Bar, thus creating a magnetic field inside the Hall Bar, resulting in a voltage between the two knobs of the Hall Bar, due to the Hall Effect. The width of the bar is 50 nm. Yet, the simulation model was built up in a way that allows for quick changes in all dimensions.

### 2.3. Simulation Considerations

**Figure 4:**
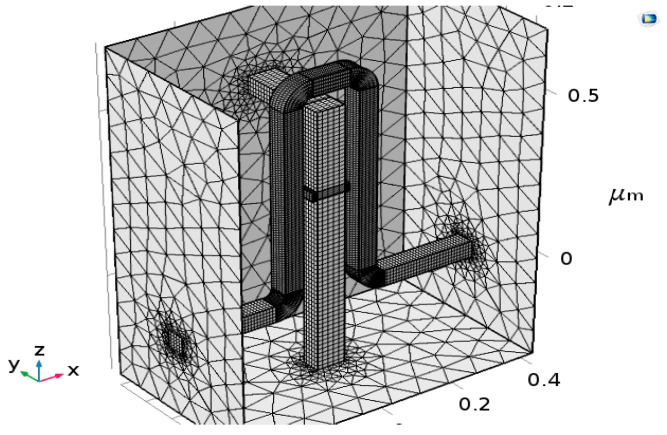
Mesh for three-dimensional (3D) simulation set-up.

**Figure 5:**
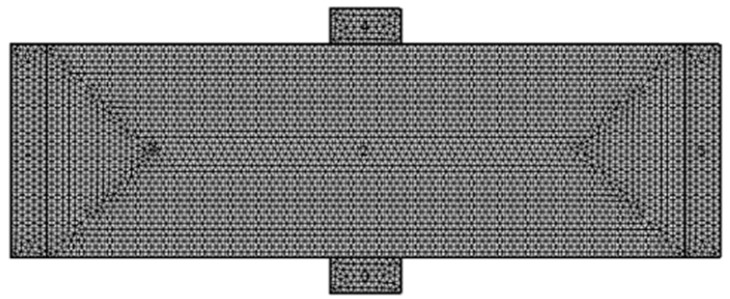
Mesh for two-dimensional (2D) simulation of the Hall Bar.

The design and the simulations of all the devices in the research group are performed while using the Comsol Multi-Physics Software Package [14]. In this specific case, the required simulation models are the AC/DC module and the semiconductors module. As shown in Figure 3, the materials that were simulated in this model are metals with high conductivity in the front panel. In addition to Comsol, Matlab software [15] for mathematical modeling of the device behavior was also used. Figure 4 and Figure 5 present the mesh considerations.

**Figure 6:**
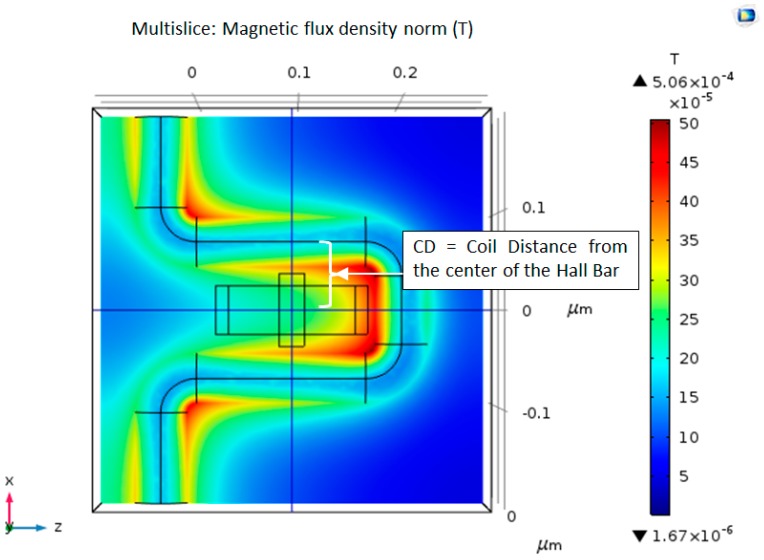
Magnetic flux density norm (T) produced by 30 nm copper coil at a of distance CD = 70 nm, and with electric current input of 30 µA.

**Figure 7:**
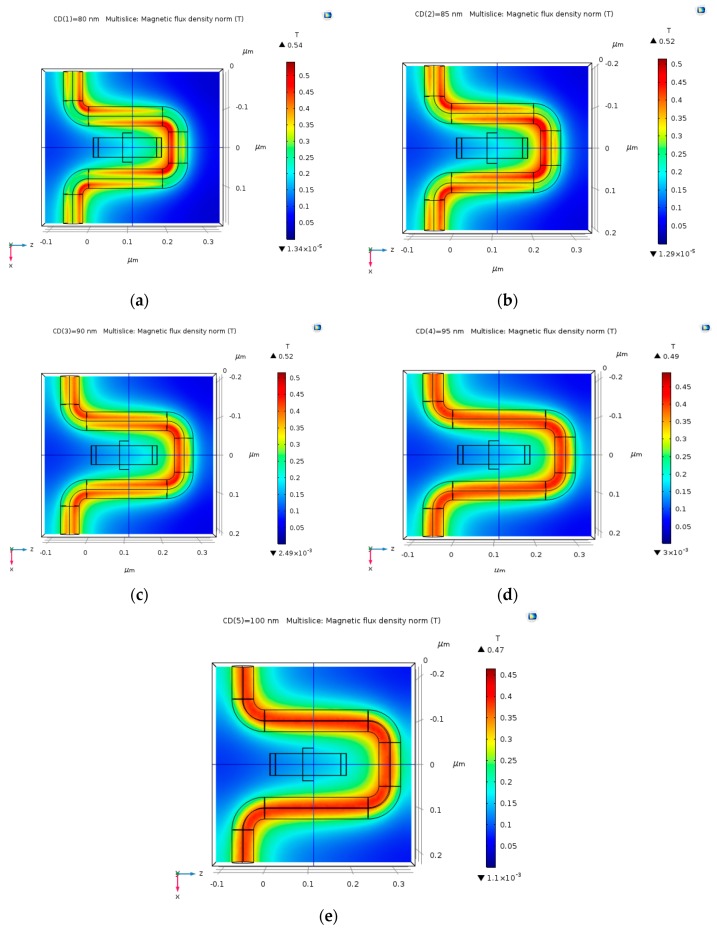
Magnetic flux density norm (T) produced by 25 nm copper coil at varying distance CD, and this time with an electric current input of 30 mA. (**a**) CD = 80 nm; (**b**) CD = 85 nm; (**c**) CD = 90 nm; (**d**) CD = 95 nm; and, (**e**) CD = 100 nm.

**Figure 8:**
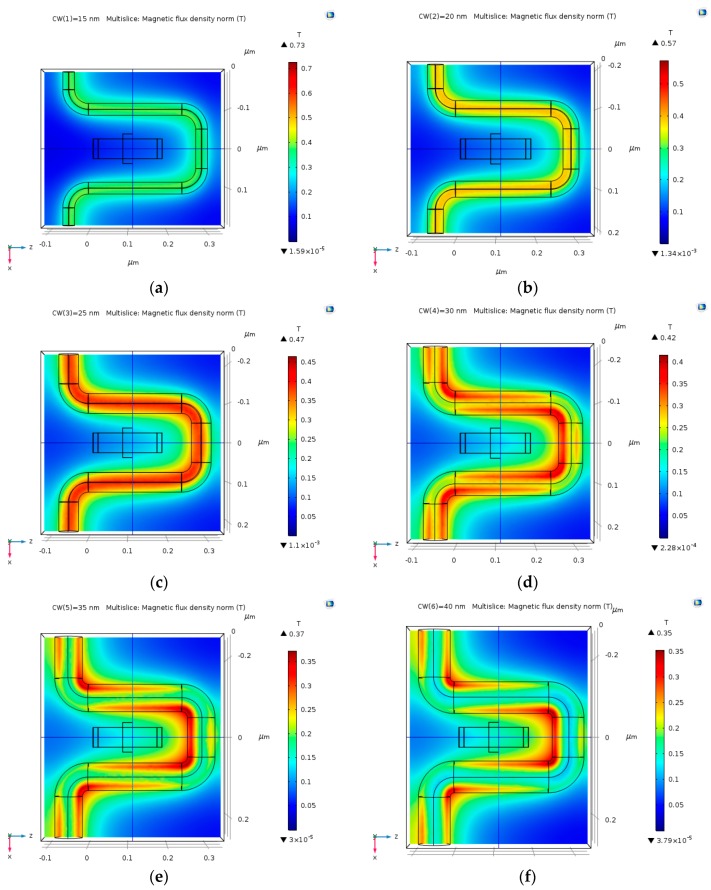
Magnetic flux density norm (T), produced by several lengths of copper coil at a distance of CD = 100 nm, and with an electric current input of 30 mA. (**a**) 15 nm copper coil; (**b**) 20 nm copper coil; (**c**) 25 nm copper coil; (**d**) 30 nm copper coil; (**e**) 35 nm copper coil; and, (**f**) 40 nm copper coil.

**Figure 9:**
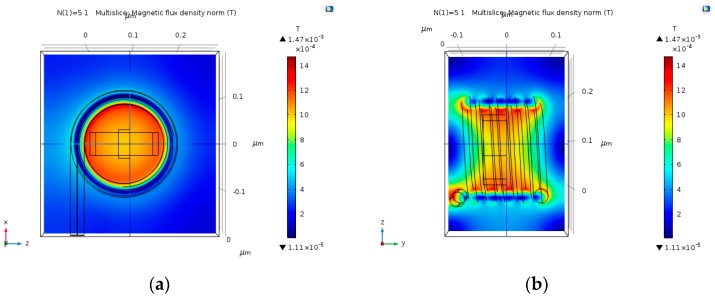
Magnetic flux density norm (T), produced by 15 nm copper coil with five loops, and an input electric current of 30 µA. (**a**) Face-view; and, (**b**) Cross-view.

**Figure 10:**
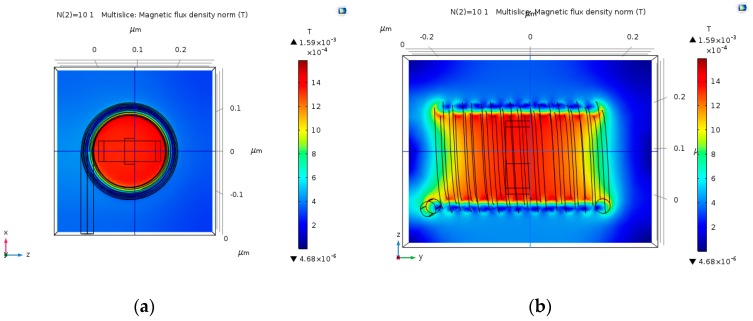
Magnetic flux density norm (T), produced by 15 nm copper coil with 10 loops, and an input electric current of 30 µA. (**a**) Face-view; and, (**b**) Cross-view.

**Figure 11:**
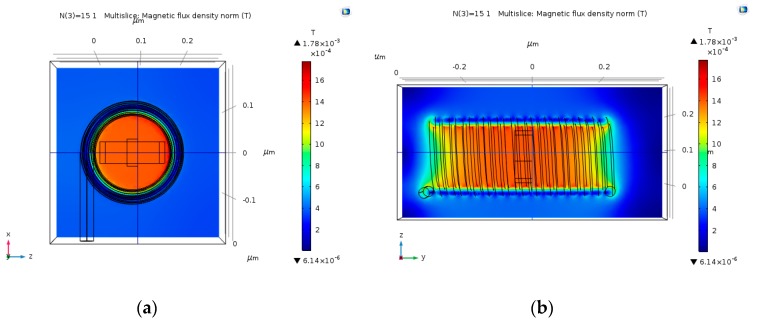
Magnetic flux density norm (T), produced by 15 nm copper coil with 15 loops, and an input electric current of 30 µA. (**a**) Face-view; and, (**b**) Cross-view.

**Figure 12:**
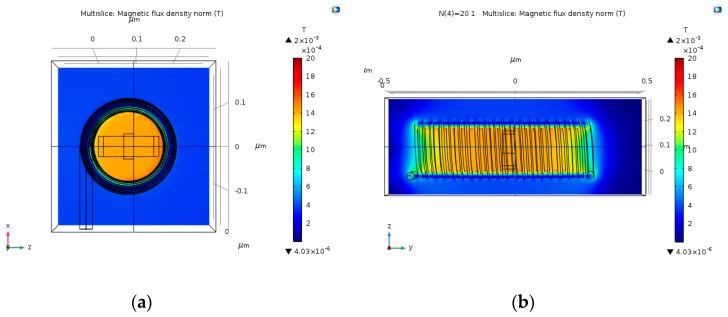
Magnetic flux density norm (T), produced by 15 nm copper coil with 20 loops, and an input electric current of 30 µA. (**a**) Face-view; and, (**b**) Cross-view.

**Figure 13:**
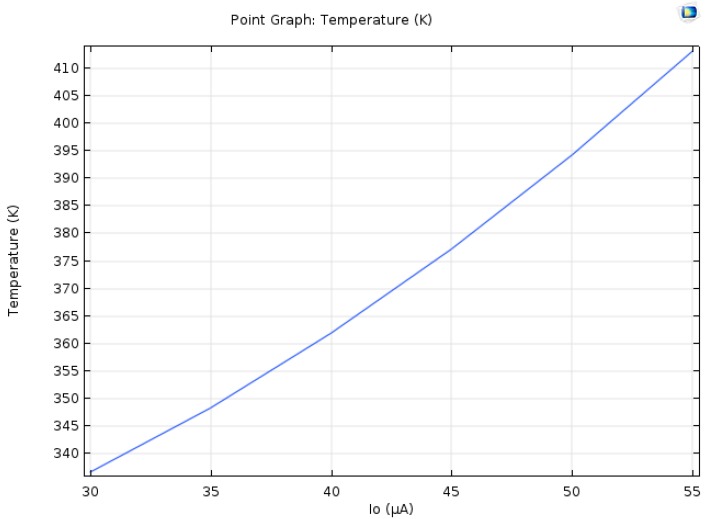
The temperature as a function of the input electric current.

**Figure 14:**
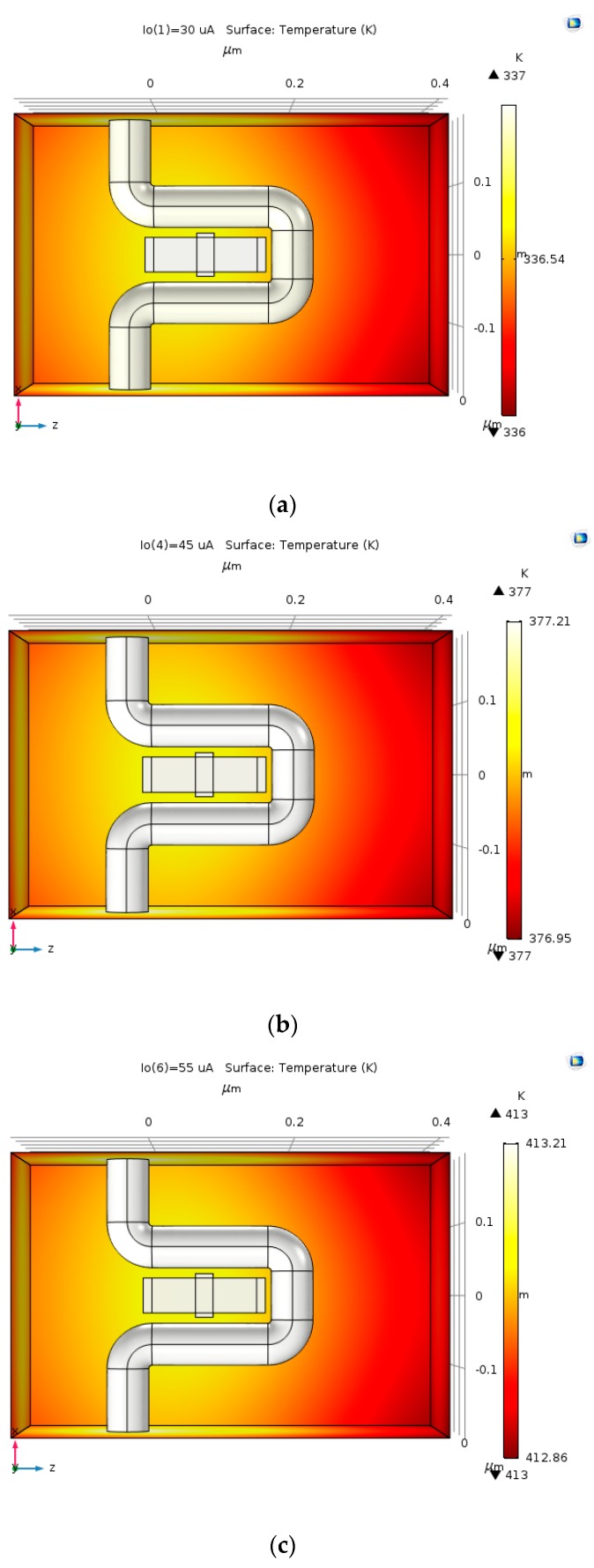
The temperature as a function of the input electric current. The temperature with input electric current: (**a**) 30 µA; (**b**) 45 µA; and, (**c**) 55 µA.

**Figure 15:**
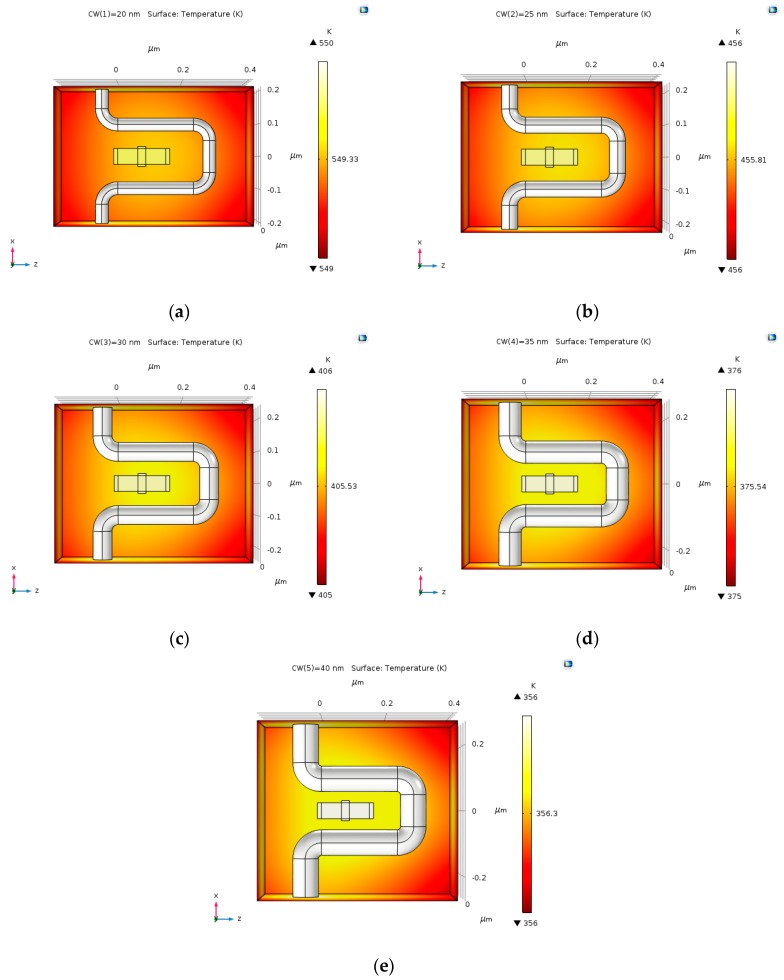
The temperature produced by varying lengths of copper coil and input electric current of 30 µA: (**a**) 20 nm; (**b**) 25 nm; (**c**) 30 nm; (**d**) 35 nm; and, (**e**) 40 nm.

**Figure 16:**
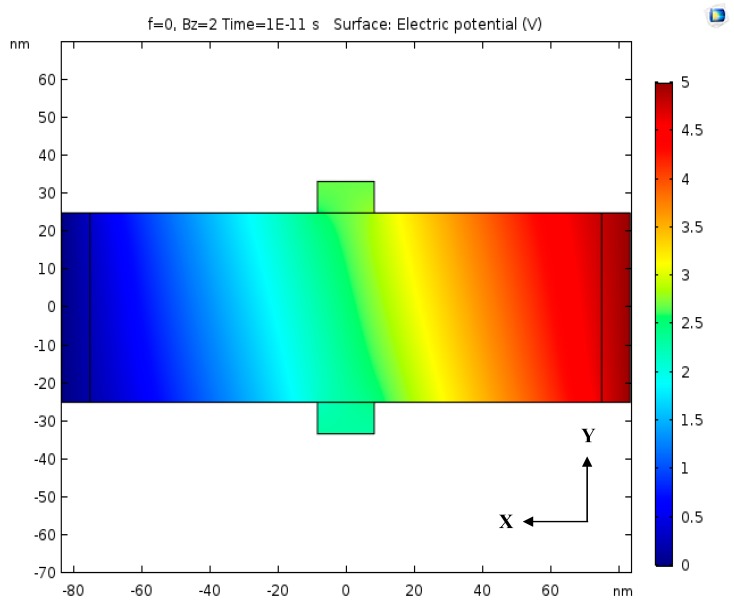
Simulation of the voltage difference across the Hall Bar that is caused by the perpendicular magnetic field.

**Figure 17:**
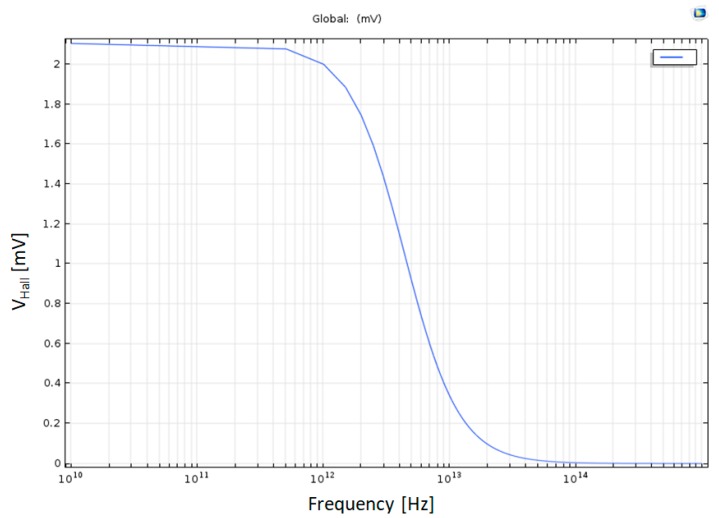
Hall Voltage as a function of the frequency in half log scale.

**Figure 18:**
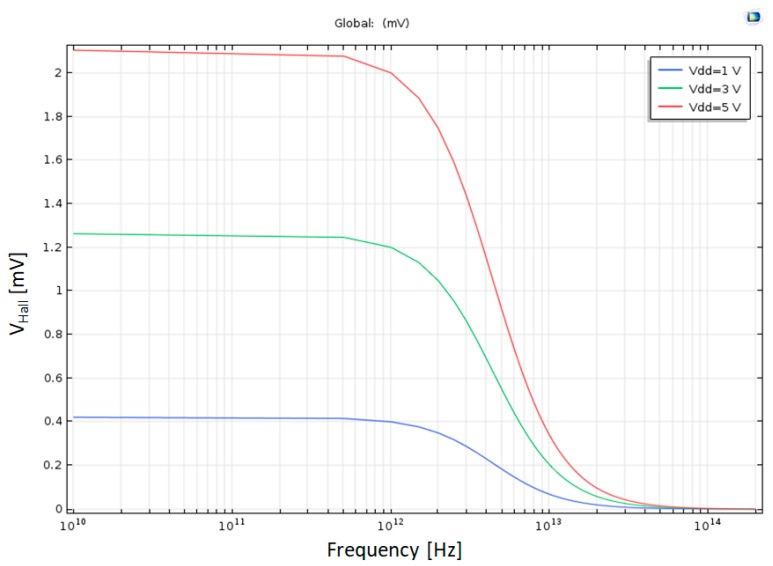
Hall Voltage as a function of the frequency in half log scale for several V_dd_ values.

**Figure 19:**
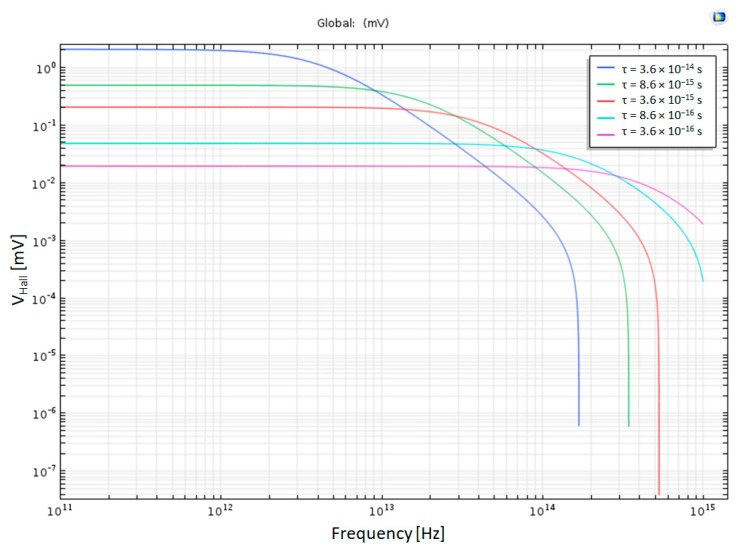
Hall Voltage as a function of the frequency for different scattering times in log-log scale.

**Figure 20:**
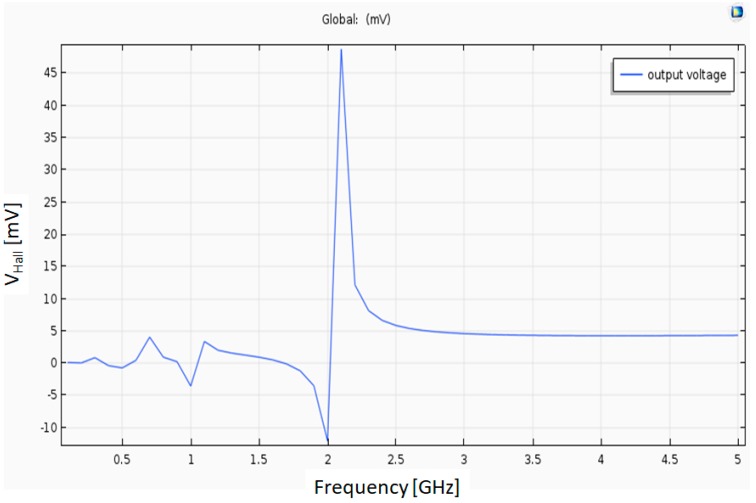
Hall Voltage as a function of the frequency for heterodyne Hall conductivity.

As presented below, in Figure 6, Figure 7, Figure 8, Figure 9, Figure 10, Figure 11, Figure 12, Figure 13, Figure 14, Figure 15, Figure 16, Figure 17, Figure 18, Figure 19 and Figure 20, tens of simulations were performed while using the Comsol Multi-Physics Package Software. It was necessary to combine the usage of several modules in order to simulate such complex analyses. The first module used is the AC/DC, whose features are the (1) Magnetic and Electric Fields (MEF), providing the magnetic field by using Ampere’s Law, and electric field by Current conservation, and the (2) Electric Currents (EC), providing the current conservation law and electric potential. The second module that used for the analyses is the Heat Transfer one, whose main features are the electric current and heat transfer providing the joule heating process.

### 2.4. Mesh Accuracy and Considerations

The geometries of the Hall Bar and the coil are discretized in squares to achieve the most accurate results for their block symmetry. The discretization of the Hall Bar’s output knobs is denser to obtain more accurate results. The remaining geometry is discretized in tetrahedron, which is the default mesh. The mesh used in the 2D simulation is the default mesh, and it is different from the 3D one. The chosen element size option was “extra fine”.

## 3. Analytical Model

### 3.1. Classical Hall Effect

The following are well-known equations and assumptions that serve as the mathematical basis to formulate the classical Hall Effect, when compared to updated models, and nanoscale considerations developed later on in this study. It is necessary to return to the electromagnetism basics before analyzing more complex case studies. Assuming:

(1) $${\vec{F}}_{\mathrm{magnetic}\;}={\vec{F}}_{\mathrm{electric}},$$

Subsequently, we get:

(2) $${q\vec{E}}_{Y}={\;q\vec{v}}_{D}\times {\vec{B}}_{Z},$$

Also:

(3) $$||{\vec{E}}_{Y}||=||{\vec{v}}_{D}||||{\vec{B}}_{Z}||$$

Since:

(4) $$V_{Y}=E_{Y}\cdot L_{y}$$

We obtain:

(5) $$V_{H}=v_{D}B_{Z}L_{y}$$

Since:

(6) $$v_{D}=\frac{I_{x}}{A_{x}*e*n}$$

We get:

(7) $$V_{H}=\frac{I_{x}{*B}_{Z}{*L}_{y}}{A_{x}*e*n}$$

(8) $$R_{\mathrm{xy}}=\;\frac{V_{Y}}{I_{x}}={\;R}_{H}B_{Z}\frac{L_{y}}{A_{x}}$$

(9) $$R_{H}=\frac{1}{\mathrm{ned}}$$

where: $L_{y}$ is the transverse width of the Hall Bar, $A_{x}$ is the transverse cross sectional area of Hall Bar, $v_{D}$ is the drift velocity, $B_{Z}$ is the magnetic field in the Z direction, $E_{Y}$ is the electric field in the Y direction, $V_{H}$ is the Hall Voltage, $V_{Y}$ is the voltage in the Y direction, $I_{x}$ is the electric current in the Hall Bar, e is the electron charge (1.6 × 10^−19^ C), n is the particle density, and $R_{H}\;$is the 3D Hall coefficient.

### 3.2. DC Hall Magneto-Resistance

The Drude model [16] provides an estimate for the resistance. This model can be applied where a precise understanding of scattering is not required. The equation of motion for the momentum per electron is as follows:

(10) $$\frac{\mathrm{dp}\left(t\right)}{\mathrm{dt}}=-\frac{p\left(t\right)}{\tau }+F\left(t\right)$$

where: p = momentum per electron, τ = collision time (mean free time), and F = external force.

The Hall Effect changes the electrical resistance of the material by using externally-applied magnetic field. Initially, the velocity of the electrons is:

(11) $$\vec{v}=v_{x}\hat{x}+v_{y}\hat{y}+v_{z}\hat{z}$$

The electric field is:

(12) $$\vec{E}=E_{x}\hat{x}$$

and the magnetic field is:

(13) $$\vec{B}=B_{z}\hat{z}$$

Accordingly, the equation of motion is:

(14) $$m_{e}\left(\frac{d}{\mathrm{dt}}+\frac{1}{\tau }\right)\vec{v}=-e\left(\vec{E}+\vec{v}\times \vec{B}\right)$$

where $m_{e}$ is the effective mass of the electron.

In the case of DC electrical conductivity, we obtain the two following equations:

(15) $$F_{x}=m_{e}\left(\frac{d}{\mathrm{dt}}+\frac{1}{\tau }\right)v_{x}=-e\left(E_{x}+v_{y}B_{z}\right)$$

(16) $$F_{y}=m_{e}\left(\frac{d}{\mathrm{dt}}+\frac{1}{\tau }\right)v_{y}={\mathrm{ev}}_{x}B_{z}$$

In steady state condition:

(17) $$\frac{m_{e}v_{x}}{\tau }=-e\left(E_{x}+v_{y}B_{z}\right)\to v_{x}=-\frac{e\tau E_{x}}{m_{e}}-\omega_{c}v_{y}\tau$$

(18) $$\frac{m_{e}v_{y}}{\tau }=-e\left(E_{y}-v_{x}B_{z}\right)\to v_{y}=-\frac{e\tau E_{y}}{m_{e}}+\omega_{c}v_{x}\tau$$

where:

(19) $$\omega_{c}=\frac{{\mathrm{eB}}_{z}}{m_{e}}$$

is the cyclotron frequency.

In steady state, since:

(20) $$v_{y}=0$$

Subsequently:

(21) $$E_{y}=m_{e}\frac{\omega_{c}v_{x}}{e}$$

(22) $$E_{x}=-m_{e}\frac{v_{x}}{e\tau }$$

Therefore:

(23) $$E_{y}=-\omega_{c}{\tau E}_{x}$$

Since:

(24) $$j_{x}=-{\mathrm{nev}}_{x}\to j_{x}=\sigma_{0}E_{x}=-\frac{{\mathrm{ne}}^{2}\tau }{m_{e}}E_{x}$$

The two-dimensional (2D)-Hall coefficient is defined as:

(25) $$R_{H}=\frac{E_{y}}{j_{x}B_{z}}=-\frac{\frac{{\mathrm{eB}}_{z}\tau }{m_{e}}E_{x}}{\frac{{\mathrm{ne}}^{2}\tau }{m_{e}}E_{x}B_{z}}=-\frac{1}{\mathrm{ne}}$$

The Hall magneto-resistance is defined as:

(26) $$R_{\mathrm{xy}}=\frac{V_{y}}{I_{x}}=R_{H}B_{z}$$

meaning that the resistance does not depend on the shape of the Hall Bar.

In the presence of magnetic field, the resistivity tensor becomes:

(27) $$\left(\begin{array}{c} E_{x}\\ E_{y} \end{array}\right)=\left[\begin{array}{cc} \rho_{\mathrm{xx}} & \rho_{\mathrm{xy}}\\ \rho_{\mathrm{yx}} & \rho_{\mathrm{yy}} \end{array}\right]\left(\begin{array}{c} j_{x}\\ j_{y} \end{array}\right)$$

(28) $$\{\begin{array}{c} \sigma_{0}E_{x}=j_{x}+\omega_{c}\tau j_{y}\\ \sigma_{0}E_{y}=-\omega_{c}\tau j_{x}+j_{y} \end{array}$$

Therefore:

(29) $$\left(\begin{array}{c} E_{x}\\ E_{y} \end{array}\right)=\left[\begin{array}{cc} \frac{1}{\sigma_{0}} & \frac{\omega_{c}\tau }{\sigma_{0}}\\ -\frac{\omega_{c}\tau }{\sigma_{0}} & \frac{1}{\sigma_{0}} \end{array}\right]\left(\begin{array}{c} j_{x}\\ j_{y} \end{array}\right)$$

(30) $$\rho_{\mathrm{xx}}=\rho_{\mathrm{yy}}=\frac{1}{\sigma_{0}}=\frac{m_{e}}{{\mathrm{ne}}^{2}\tau }$$

(31) $$\rho_{\mathrm{xy}}=-\rho_{\mathrm{yx}}=\frac{\omega_{c}\tau }{\sigma_{0}}=\frac{B_{z}}{\mathrm{ne}}$$

The conductivity tensor is:

(32) $$\left(\begin{array}{c} j_{x}\\ j_{y} \end{array}\right)=\left[\begin{array}{cc} \sigma_{\mathrm{xx}} & \sigma_{\mathrm{xy}}\\ \sigma_{\mathrm{yx}} & \sigma_{\mathrm{yy}} \end{array}\right]\left(\begin{array}{c} E_{x}\\ E_{y} \end{array}\right)$$

Therefore:

(33) $$\left[\begin{array}{cc} \sigma_{\mathrm{xx}} & \sigma_{\mathrm{xy}}\\ \sigma_{\mathrm{yx}} & \sigma_{\mathrm{yy}} \end{array}\right]={\left[\begin{array}{cc} \rho_{\mathrm{xx}} & \rho_{\mathrm{xy}}\\ \rho_{\mathrm{yx}} & \rho_{\mathrm{yy}} \end{array}\right]}^{-1}$$

where:

(34) $$\sigma_{\mathrm{xx}}=\sigma_{\mathrm{yy}}=\frac{\rho_{\mathrm{xx}}}{\rho_{\mathrm{xx}}{}^{2}+\rho_{\mathrm{xy}}{}^{2}}=\frac{\sigma_{0}}{1+{\left(\omega_{c}\tau \right)}^{2}}=\frac{\sigma_{0}}{1+{\left(\sigma_{0}R_{H}B_{z}\right)}^{2}}$$

(35) $$\sigma_{\mathrm{xy}}=-\sigma_{\mathrm{yx}}=-\frac{\rho_{\mathrm{xy}}}{\rho_{\mathrm{xx}}{}^{2}+\rho_{\mathrm{xy}}{}^{2}}=-\frac{\sigma_{0}\omega_{c}\tau }{1+{\left(\omega_{c}\tau \right)}^{2}}=\frac{\sigma_{0}{}^{2}R_{H}B_{z}}{1+{\left(\sigma_{0}R_{H}B_{z}\right)}^{2}}$$

### 3.3. Approximation to the Dynamic Magneto-Conductivity Tensor for Free Electrons

At this stage, it was necessary to develop a new approach for a specific case study of weak magnetic field acting on a small input signal, which means creating small amplification. The Dynamic magneto-conductivity tensor for free electrons in the case of oscillating magnetic field in the Z direction, such as $\vec{B}\left(t\right)=B_{0}e^{-i\omega t}\hat{z}$, and static electric field in the X direction, will be calculated by using Perturbation Theory. Additionally, this model uses the assumptions of the Drude model as shown in the DC Hall magneto-resistance section above, therefore the ballistic conductance is not taken into account in this model. Moreover, this model does not include quantum hall effect, which becomes relevant for strong magnetic fields B > 0.5 T. The equations of motion, for the momentum per electron, are as follows:

(36) $$m_{e}\left(\frac{d\vec{v}}{\mathrm{dt}}+\frac{\vec{v}}{\tau }\right)=-e\left(\vec{E}+\varepsilon \vec{v}\times \vec{B}\right)$$

where

(37) $$\vec{v}={\vec{v}}_{0}+{\varepsilon \vec{v}}_{1}+\varepsilon^{2}{\vec{v}}_{2}$$

and

(38) $$\varepsilon =\frac{||{\vec{v}}_{0}||||\vec{B}||}{||\vec{E}||}\ll 1$$

For zero order $\varepsilon^{0}$, the equation becomes:

(39) $$m_{e}\left(\frac{{d\vec{v}}_{0}}{\mathrm{dt}}+\frac{{\vec{v}}_{0}}{\tau }\right)=-e\vec{E}$$

Since $\vec{E}$ is static, we get

(40) $$\frac{{d\vec{v}}_{0}}{\mathrm{dt}}=0$$

Therefore:

(41) $$m_{e}\frac{{\vec{v}}_{0}}{\tau }=-e\vec{E}\to {\vec{v}}_{0}=\;\frac{-e\tau }{m_{e}}\vec{E}\to {\vec{v}}_{0}=\;-\mu_{e}\vec{E}$$

From that, we get:

(42) $$\varepsilon =\frac{||{\vec{v}}_{0}||||\vec{B}||}{||\vec{E}||}={µ}_{e}||\vec{B}||$$

Since

J = −nev(43)

(44) $${\vec{J}}_{0}=\frac{{\mathrm{ne}}^{2}\tau }{m_{e}}\vec{E}={\;\sigma }_{0}\vec{E}$$

For zero order $\varepsilon^{1}$ the equation becomes:

(45) $$m_{e}\left(\frac{{d\vec{v}}_{1}}{\mathrm{dt}}+\frac{{\vec{v}}_{1}}{\tau }\right)=-{e\vec{v}}_{0}\times \vec{B}$$

Since

(46) $$\vec{B}\left(t\right)={\vec{B}}_{0}e^{-i\omega t},\;{\vec{v}}_{1}={\vec{v}}_{10}e^{-i\omega t}$$

Therefore:

(47) $$m_{e}\left(-j\omega +\frac{1}{\tau }\right){\vec{v}}_{10}e^{-i\omega t}=-{e\vec{v}}_{0}\times {\vec{B}}_{0}e^{-i\omega t}$$

Since

(48) $${\vec{v}}_{0}=-\mu_{e}\vec{E}$$

(49) $$\left(1-j\omega \tau \right){\vec{v}}_{10}=\frac{e\tau \mu_{e}}{m_{e}}\vec{E}\times {\vec{B}}_{0}\to {\vec{v}}_{10}=\frac{\mu_{e}{}^{2}\vec{E}\times {\vec{B}}_{0}}{\left(1-j\omega \tau \right)}$$

Since

(50) J = −nev

(51) $${\vec{J}}_{10}=\frac{\sigma_{0}\mu_{e}{\vec{B}}_{0}\times \vec{E}}{\left(1-j\omega \tau \right)}=\left[\begin{array}{ccc} 0 & -\frac{\sigma_{0}\mu_{e}||\vec{B}||}{1-j\omega \tau } & 0\\ \frac{\sigma_{0}\mu_{e}||\vec{B}||}{1-j\omega \tau } & 0 & 0\\ 0 & 0 & 0 \end{array}\right]\vec{E}$$

(52) $${\vec{J}}_{10}=\left[\begin{array}{ccc} 0 & -\frac{\sigma_{0}\varepsilon }{1-j\omega \tau } & 0\\ \frac{\sigma_{0}\varepsilon }{1-j\omega \tau } & 0 & 0\\ 0 & 0 & 0 \end{array}\right]\vec{E}$$

For zero order $\varepsilon^{2}$ the equation becomes:

(53) $$m_{e}\left(\frac{{d\vec{v}}_{2}}{\mathrm{dt}}+\frac{{\vec{v}}_{2}}{\tau }\right)=-{e\vec{v}}_{1}\times \vec{B}$$

Since

(54) $${\vec{v}}_{1}\vec{B}={\vec{v}}_{10}{\vec{B}}_{0}e^{-i2\omega t}$$

and

(55) $${\vec{v}}_{2}={\vec{v}}_{20}e^{-i2\omega t}$$

Therefore

(56) $$m_{e}\left(-j2\omega +\frac{1}{\tau }\right){\vec{v}}_{20}e^{-i2\omega t}=-{e\vec{v}}_{10}\times {\vec{B}}_{0}e^{-i2\omega t}$$

Since

(57) $${\vec{v}}_{10}=\frac{\mu_{e}{}^{2}\vec{E}\times {\vec{B}}_{0}}{\left(1-j\omega \tau \right)}$$

Subsequently

(58) $$\left(1-j2\omega \tau \right){\vec{v}}_{20}=-\frac{e\tau }{m_{e}}\frac{\mu_{e}{}^{2}\left(\vec{E}\times {\vec{B}}_{0}\right)}{\left(1-j\omega \tau \right)}\times {\vec{B}}_{0}=-\frac{e\tau }{m_{e}}\frac{\mu_{e}{}^{2}{\vec{B}}_{0}}{\left(1-j\omega \tau \right)}\times \left({\vec{B}}_{0}\times \vec{E}\right)$$

Since

(59) J = −nev

Subsequently

(60) $${\vec{J}}_{20}=\frac{\sigma_{0}\mu_{e}{}^{2}{\vec{B}}_{0}}{\left(1-j\omega \tau \right)\left(1-j2\omega \tau \right)}\times \left({\vec{B}}_{0}\times \vec{E}\right)$$

(61) $${\vec{J}}_{20}=\frac{\sigma_{0}\mu_{e}{}^{2}||{\vec{B}}_{0}||{}^{2}}{\left(1-j\omega \tau \right)\left(1-j2\omega \tau \right)}\left[\begin{array}{ccc} 0 & -1 & 0\\ 1 & 0 & 0\\ 0 & 0 & 0 \end{array}\right]\left[\begin{array}{ccc} 0 & -1 & 0\\ 1 & 0 & 0\\ 0 & 0 & 0 \end{array}\right]\vec{E}$$

(62) $${\vec{J}}_{20}=\frac{\sigma_{0}\mu_{e}{}^{2}||{\vec{B}}_{0}||{}^{2}}{\left(1-j\omega \tau \right)\left(1-j2\omega \tau \right)}\left[\begin{array}{ccc} -1 & 0 & 0\\ 0 & -1 & 0\\ 0 & 0 & 0 \end{array}\right]\vec{E}$$

(63) $${\vec{J}}_{20}=\frac{\sigma_{0}\varepsilon^{2}}{\left(1-j\omega \tau \right)\left(1-j2\omega \tau \right)}\left[\begin{array}{ccc} -1 & 0 & 0\\ 0 & -1 & 0\\ 0 & 0 & 0 \end{array}\right]\vec{E}$$

Since

(64) $$\vec{J}={\vec{J}}_{0}+{\vec{J}}_{1}+{\vec{J}}_{2}$$

and

(65) $$\vec{J}=\left[\overset{↔}{\sigma }\right]\vec{E}$$

(66) $$\vec{J}=\left[\begin{array}{ccc} \sigma_{0}-\frac{\sigma_{0}\varepsilon^{2}}{\left(1-j\omega \tau \right)\left(1-j2\omega \tau \right)} & -\frac{\sigma_{0}\varepsilon }{1-j\omega \tau } & 0\\ \frac{\sigma_{0}\varepsilon }{1-j\omega \tau } & \sigma_{0}-\frac{\sigma_{0}\varepsilon^{2}}{\left(1-j\omega \tau \right)\left(1-j2\omega \tau \right)} & 0\\ 0 & 0 & \sigma_{0} \end{array}\right]\vec{E}$$

(67) $$\left[\begin{array}{ccc} \sigma_{\mathrm{xx}} & \sigma_{\mathrm{xy}} & \sigma_{\mathrm{xz}}\\ \sigma_{\mathrm{yx}} & \sigma_{\mathrm{yy}} & \sigma_{\mathrm{yz}}\\ \sigma_{\mathrm{zx}} & \sigma_{\mathrm{zy}} & \sigma_{\mathrm{zz}} \end{array}\right]=\left[\begin{array}{ccc} \sigma_{0}-\frac{\sigma_{0}\varepsilon^{2}}{\left(1-j\omega \tau \right)\left(1-j2\omega \tau \right)} & -\frac{\sigma_{0}\varepsilon }{1-j\omega \tau } & 0\\ \frac{\sigma_{0}\varepsilon }{1-j\omega \tau } & \sigma_{0}-\frac{\sigma_{0}\varepsilon^{2}}{\left(1-j\omega \tau \right)\left(1-j2\omega \tau \right)} & 0\\ 0 & 0 & \sigma_{0} \end{array}\right]$$

### 3.4. Heterodyne Hall Effect in a Two-Dimensional Electron Gas

The Hall Effect with a magnetic and electric field oscillating in time with resonant frequencies is a phenomenon that realizes an example of heterodyne device with the magnetic field acting as a driving and it is analyzed in detail in its classical versions while using Floquet theory [17]. A bulk current flowing perpendicularly to the applied electric field is found, with a frequency shifted by integer multiples of the driving frequency. The heterodyne conductivity [17] $\sigma_{\mathrm{ab}}^{m,n}$, introduced here for classical case, is a four index tensor implicitly that is defined by the linear relation that holds between the electric current density $j_{a}\left(mΩ\right)$ of the output signal with frequency mΩ, flowing along the a-direction (a, b = x, y, z) and the weak electric field $E_{b}^{n}$ along the b-direction with frequency nΩ.

(68) $$j_{a}\left(mΩ\right)\;=\;\sum_{b}{\;\sigma }_{\mathrm{ab}}^{m,n}\;\left(nΩ\right)E_{b}^{n},$$

It can be shown that the classical case the heterodyne conductivity for n = 0 (static electric field) [17] is:

(69) $$\sigma_{\mathrm{xy}}^{0,0}=0\;,{\;\sigma }_{\mathrm{yy}}^{0,0}={\;\sigma }_{0}J_{0}{\left(r\right)}^{2},$$

(70) $$\sigma_{\mathrm{xy}}^{1,0}=\sigma_{0}Ω\tau \frac{J_{0}\left(r\right)J_{1}\left(r\right)}{1+{\left(Ω\tau \right)}^{2}}\;,{\;\sigma }_{\mathrm{yy}}^{1,0}={\;\sigma }_{0}Ω\tau \frac{J_{0}\left(r\right)J_{1}\left(r\right)}{1+{\left(Ω\tau \right)}^{2}},$$

where Ω is the magnetic field frequency, $\omega_{c}$ is the cyclotron frequency,${\;J}_{0}\left(r\right)\;$is zero order Bessel function, $J_{1}\left(r\right)\;$is first order Bessel function, and $r=\frac{\omega_{c}}{Ω}$ is the Bessel function argument.

### 3.5. Dielectric Tensor for Free Electron Model

From the Maxwell equation, the dielectric function tensor of the medium is related to the conductivity tensor, as:

(71) $$\varepsilon =1+j\left(\frac{4\pi }{\varepsilon_{0}\omega }\right)\sigma ,$$

Therefore

(72) $$\;\varepsilon =\left[\begin{array}{ccc} 1+\frac{4\pi j}{\varepsilon_{0}\omega }\sigma_{\mathrm{xx}} & \frac{4\pi j}{\varepsilon_{0}\omega }\sigma_{\mathrm{xy}} & 0\\ \frac{4\pi j}{\varepsilon_{0}\omega }\sigma_{\mathrm{yx}} & 1+\frac{4\pi j}{\varepsilon_{0}\omega }\sigma_{\mathrm{yy}} & 0\\ 0 & 0 & 1+\frac{4\pi j}{\varepsilon_{0}\omega }\sigma_{\mathrm{zz}} \end{array}\right],$$

## 4. Results

### 4.1. Parameters

Preliminary results were obtained. The parameters used for the 2D simulations are presented in Table 1.

### 4.2. Single Loop Copper Wire Magnetic Field

As the electric current goes through the copper coil, it creates a magnetic field around the coil by Ampere’s Law. Figure 6 shows the magnetic flux density norm (T) is distributed in our geometry. The direction of the magnetic field goes into the Hall Bar. The simulations were conducted with input electric current of 30 µA and 30 mA, called I0 which is the DC input current inserted in Comsol calculations, and which will serve later as the amplitude of an AC input signal. Different copper coil radii were chosen, ranging from 15 nm to 40 nm (CW). Additionally, the distance between the center of the Hall Bar to the center of the coil varies between 70 nm and 100 nm (CD). As predicted, the magnetic field is stronger as the coil distance (CD) gets smaller, as demonstrated in Figure 7a–e. The max magnetic field varies between 0.47 T and 0.54 T. The magnetic field around the Hall Bar is between 0.2 T and 0.25 T.

The magnetic field around the Hall Bar is stronger as the coil radius (CW) increases, as shown in following Figure 8a–f. The magnetic field around the Hall Bar is between 0.1 T and 0.2 T.

### 4.3. Multi-Loop Copper Wire Magnetic Field

Multi-loop coil could potentially produce stronger magnetic fields, thus obtaining stronger Hall Voltage output. The input electric current that was used in these simulations is 30 µA. As predicted, the magnetic field gets stronger as the number of loops increases (Figure 9, Figure 10, Figure 11 and Figure 12). The magnetic field is more uniform around the Hall Bar than it was in the single loop coil. The max magnetic field is in the range of 1.49 mT to 2 mT. In case the input electric current becomes 30 mA (instead of 30 µA), the maximal value of the magnetic field will be in the range of 1.49 T to 2 T. The magnetic field becomes more uniform as the number of loops increases.

### 4.4. Heat Transfer of the Device

Electric current through a wire produces heat by joule heating. Temperature adds more noise to the system, and the goal in these simulations is to find the temperature around the Hall Bar. The Hall Bar in these simulations is made of GaAs. The heat transfer coefficient that was used in these simulations is 100 $\frac{W}{m^{2}K}$, as shown in Figure 13. It is the heat transfer coefficient for forced convection; moderate speed flow of air over a surface [19]. The dimensions of the HAND for these simulations are as shown in Figure 6.

As shown in Figure 13 and Figure 14, as the current gets bigger the temperature gets higher. The temperature ranges between 337 K and 413 K. If the input electric current is 30 mA–50 mA the temperature could elevate to two to three orders of magnitude higher than the current simulation; therefore, cooling is required to make the HAND feasible.

### 4.5. Current Density Effect on Joule Heating

As the current density becomes higher, the heat that is produced by joule heating increases. As predicted, the temperature declines as the current density decreases, as shown in Figure 15a–e.

### 4.6. Hall Bar 2D AC/DC Simulations

In the 2D simulation, the magnetic field that was used was defined by $\vec{B}\left(t\right)=B_{z}e^{-i\omega t}\hat{z}$. This magnetic field should be the same magnetic field that could be produced by AC electric current through the copper coil. The magnetic field that was produced by the electric current in the 3D simulation was not used in the 2D simulation. This magnetic field $\vec{B}\left(t\right)$ is perpendicular to the Hall Bar. In the Hall Bar there is a DC electric current $I_{x}$ in the X direction, so the Hall Effect produces a Hall Voltage between the two output knobs of the Hall Bar, as shown in Figure 16. In the DC magnetic field, the output voltage is highest in steady state, when there is no electric current $I_{y}$ in the Y direction.

### 4.7. Frequency Effect on Hall Voltage

The Hall Voltage stays constant V_Hall_ = 2.1 mV until f = 400 GHz, and then it starts to decrease to zero around. f = 8.10^13^ Hz, as shown in Figure 17.

### 4.8. The Effect of the Applied Voltage V_dd_ on Hall Voltage

A higher V_dd_ means a stronger electric field in the X direction, which causes higher electric current in the X direction. If this current increases in DC, the equilibrium between the Lorentz force (magnetic field) to the Coulomb force (electric field in Y direction) will be achieved at a higher Hall Voltage. The same happens in AC, as V_dd_ gets bigger; the Hall Voltage between the Hall Bar knobs increases, as shown in Figure 18.

### 4.9. The Effects of Scattering Time on the Hall Voltage

This simulation depicts the effects of scattering time on the frequency response; therefore, different scattering times were chosen. In reality, different materials will be chosen or different doping in each material, which will change the scattering time due to impurities scattering, as can be seen by Matthiessen’s rule:

(73) $$\frac{1}{\tau }=\frac{1}{\tau_{\mathrm{defects}}}+\frac{1}{\tau_{\mathrm{lattice}}}+\frac{1}{\tau_{\mathrm{impurities}}}+\cdots$$

The Hall Bar is made of Copper in this simulation.

It is important to note that this simulation uses the Drude model for free electron and it does not take the ballistic effect or quantum effects into account.

The assumption that was used in this simulation is that the scattering time τ is proportional to the electron mobility and the electrical conductivity, meaning:

(74) $$\sigma_{0}\propto \mu_{e}\propto \tau$$

This means that as the scattering time decreases the electron mobility and the electrical conductivity decreases. As τ gets lower, the device can work for higher frequencies, but the overall Hall Voltage also decreases. For τ = 0.36 × 10^−16^ s, the HAND can work at frequencies up to f = 200 THz, but the Hall Voltage is much smaller than in the case of τ = 3.6 × 10^−14^ s, as shown in Figure 19.

### 4.10. Heterodyne Hall Effect Simulation

In conclusion, we obtained preliminary results for this simulation, but more research is required. In this simulation, the Hall Bar is doped Silicon. In Figure 20, there appears to be resonant frequency around 2.1 GHz where the output voltage is 45 mV. The HAND’s behavior in this model is different when compared to the approximation that was made in the dynamic magneto-resistance model. Additionally, it seems that the HAND acts as high pass filter (HPF) in this physical model (see Figure 20). The output voltage stabilizes around 5 mV after the resonant frequency.

## 5. Discussion

### 5.1. Accuracy

A better understanding of the physical model is required in order to achieve a more accurate simulation. In the heterodyne Hall Effect simulation, the result was different as compared to the approximation that was made in the dynamic conductivity model. More research is required for a better understanding of the heterodyne model. Moreover, the quantum Hall Effect model might be required to receive accurate results.

In the other simulations, the Hall Bar is made of copper and the scattering time of copper is in the range of femtoseconds, meaning τ = 36 × 10^−15^ s [20], and the simulations run for lower scattering time up to τ = 0.36 × 10^−15^ s, as shown in Figure 15. The simulations show that the HAND can work for up to f = 200 THz for a lower scattering time, but as τ gets lower, the Hall Voltage, overall, also gets smaller. Hence, there is a tradeoff between higher amplification to higher frequencies. More research is required for a better understanding of this device. One can note that the required feasibility is not about the frequency response. It is quite clear that this device (like any other electronic device) works faster as we scale it down. It is our plan to continue developing the mathematical model and the simulations since a first generation of this device did not show the expected high frequencies. The question is whether we can really scale down the device that was invented 140 years ago. Almost the same material was used, as we do (high mobility III-V), but then a huge electromagnet was probably used to drive the device (completely isolated from the bar itself). We obviously cannot use such a magnet in a nano-device, so the question remains as to whether we can operate the device with a single lap electromagnet. Currently, we are quite convinced that for DC the answer is no, but in AC and using a differential mode it might work. On the chip there are devices that are designed to operate at differential mode, but we came across the isolation issue as we tried to measure it: that is, we are not sure that the input and output can share the same ground in this device (like common source in standard transistor).

Our actual perspective is that a lot of know how to this device should come from the field of superconductors (which has to be current driven), but this idea is yet to be investigated. Intuitively, with all of the low temperature electronic surrounding us for quantum computing, perhaps the right way to solve this issue is to use superconductor gate, since (as mentioned before) we think that putting the standard metal electromagnet so close to the nano-bar only modulates the nano-bar temperature (which is an interesting topic in itself).

### 5.2. High Temperature

Four possible paths are suggested in order to solve the problem of high temperature generated due to extremely high current density in the copper nano wire that surrounds the Hall Bar:

1. Optimization of the HAND size—as we increase the electromagnet cross-section area, the larger the sustained current, the stronger the magnetic field is. However, it is important to keep HAND small enough, first in order to prevent it from losing its nanoscale advantage. In such a case, the magnetic field in the Hall Bar zone may be even weaker.
2. The use of different materials—different materials may sustain higher current densities than copper, therefore it could be part of the solution to the high temperature.
3. The field of superconductors—superconductors may help in producing the magnetic field we need, this time with lower generated temperatures.
4. Changing the geometry and components of the HAND—increasing the loop density, as shown above in the multi-loop simulation, might help in increasing the magnetic field intensity with the same current density. Moreover, adding a ferromagnetic core may be a possible solution in order to concentrate the magnetic flux and create a more powerful magnet.

Other solutions to produce a stronger magnetic field more efficiently and with less current density may also exist.

## 6. Conclusions

Several aspects of a new Hall Amplifier Nanoscale Device (HAND) were proposed summarizing the main achievements of this first research, based on complementary studies and novelties: Concept, architecture, analytical adapted models, and complementary numerical simulations. If in the past ballistic deflation devices in which the deflection is achieved with electrical field were studied, we, on the other hand, explore an alternative way with magnetic fields. After simple calculations, it appears that for the allowed input current, the magnetic field is too small to be measured in DC. However AC/RF next research might bring additional understanding on how to implement and process such as device, until future integration in circuitry. Because this device is nanoscale, and the Hall Bar is made of doped GaAs, a ballistic model for the quantum Hall Effect is required. Kubo formulation [17,21] (or Kubo-Greenwood-Chester-Thellung) is perfectly suited to investigate quantum transport in disordered bulk materials, but it is incomplete to simulate nanoscale devices, especially when approaching the ballistic regime. Indeed, in such situations, contact effects and non-equilibrium transport properties are better captured by the Landauer–Büttiker formalism. Therefore, Landauer–Büttiker Formalism [21,22,23,24] should be used in future research. It is important to note that, in the current research, cooling methods were required in order to get stronger magnetic fields. The Quantum Hall Effect will not be relevant if the magnetic field will be less then B < 0.5 T [24]. The field of superconductors could possibly help to achieve a stronger magnetic field with less heat emitted by joule heating, thus enabling the relevance of the quantum Hall Effect to the HAND operation.

Regarding the prospect of further research into this topic, one of the main challenges seems to be the Joule heating that is associated with the powering of the magnetic coil. Since our current MOS Technology is limited by Joule heating of this same size, it looks difficult, at this stage, to see an adequate realizable solution. The proposed directions mentioned in the discussion may be investigated. Not only fabrication concerns may worry us, but also additional open fundamental questions may need to be addressed, as well as more complex simulation scenarii, such as running the simulations using ballistic conduction. At the end, measurement issues may also rise. For example, since everything is low impedance how to control the current flow—should there be separated ground for input and output? Complementary research will follow for sure, and we are already there.

## 7. Patents

This research is the basis for several future patents.

## Figures and Tables

**Table 1:** Hall Amplifier Nanoscale Device (HAND’s) parameters.

Parameter	Parameters Definition	Values
**Device Parameters:**		
σ_2D Cu_	Hall Bar Conductivity in 2D simulations	5.998 × 10^7^ S/m
Σ _Doped Si_	Hall Bar Conductivity in heterodyne simulation	1.04 × 10^3^ S/m
R_H_	Hall Coefficient	8.1202 × 10^11^ m^3^/(s⋅A)
WB	Hall Bar Width	50 nm
HB	Hall Bar length	150 nm
TB	Hall Bar Thickness	50 nm
µe _Cu_	Electron Mobility in Copper	48.705 cm^2^/(V⋅s)
µe _Si_	Electron Mobility in Silicon	1400 cm^2^/(V⋅s) [18]
µe GaAs	Electron Mobility in Gallium Arsenide	8500 cm^2^/(V⋅s) [18]
**Comsol Setup Used Parameters:**		
B_z_	Magnetic field in Z direction	0.3 T
Τ	Scattering Time of Copper	3.6 × 10^−14^ s
τ_T_	A variable to change the scattering time	3.6 × 10^−14^ s–3.6 × 10^−16^ s
Freq	Frequency	
τ	Scattering time of silicon	0.21 × 10^−12^ s
V_dd_	Applied Voltage	1 V–5 V
I_0_	Input electric current in the Joule heating simulation	30 µA–55 µA
**Measured Parameters:**		
|V_Hall 2D_|	Output Voltage in 2D AC/DC simulations	2.1 mV
f_c.o. Cu_	Cut off frequency in Copper	~1 THz
B	Magnetic flux density norm	Depends on simulation
T	Temperature	Depends on simulation
V_Hall Het_	Resonant Output voltage in the heterodyne simulation	45 V
